# The challenges of interventions to promote healthier food in independent takeaways in England: qualitative study of intervention deliverers’ views

**DOI:** 10.1186/s12889-018-5096-3

**Published:** 2018-01-27

**Authors:** Louis Goffe, Linda Penn, Jean Adams, Vera Araujo-Soares, Carolyn D. Summerbell, Charles Abraham, Martin White, Ashley Adamson, Amelia A. Lake

**Affiliations:** 1 Institute of Health & Society, Newcastle University, Newcastle upon Tyne, UK; 2 Human Nutrition Research Centre, Newcastle University, Newcastle upon Tyne, UK; 3 Fuse – UKCRC Centre for Translational Research in Public Health, Newcastle upon Tyne, UK; 4 Centre for Diet and Activity Research (CEDAR), MRC Epidemiology Unit, University of Cambridge, Cambridge, UK; 5 School of Applied Social Sciences, Durham University, Durham, UK; 6 Psychology Applied to Health, Medical School, University of Exeter, Exeter, UK; 7 Department of Science, School of Science, Engineering and Design, Teesside University, Middlesbrough, UK

**Keywords:** Public health nutrition, Intervention, Qualitative methods, Food outlets, Diet, Takeaways, Food environments, Health promotion

## Abstract

**Background:**

Much of the food available from takeaways, pubs and restaurants particularly that sold by independent outlets, is unhealthy and its consumption is increasing. These food outlets are therefore important potential targets for interventions to improve diet and thus prevent diet related chronic diseases. Local authorities in England have been charged with delivering interventions to increase the provision of healthy food choices in independent outlets, but prior research shows that few such interventions have been rigorously developed or evaluated. We aimed to learn from the experiences of professionals delivering interventions in independent food outlets in England to identify the operational challenges and their suggestions for best practice.

**Methods:**

We used one-to-one semi-structured qualitative interviews to explore the views and experiences of professionals who were either employees of, or contracted by, a local authority to deliver interventions to increase the provision of healthier food choices in independent food outlets. Purposive sampling was used to recruit a sample which included men and women, from a range of professional roles, across different areas of England. Interviews were informed by a topic guide, and proceeded until no new themes emerged. Interviews were recorded, transcribed verbatim and analysed using the Framework method.

**Results:**

We conducted 11 individual interviews. Participants focussed on independent takeaways and their unhealthy food offerings, and highlighted the advantages and disadvantages of intervention delivery methods, their evaluation and impact.

The main barriers to implementation of interventions in independent takeaways were identified as limited funding and the difficulties of engaging the food outlet owner/manager. Engagement was thought to be facilitated by delivering intensive, interactive and tailored interventions, clear and specific information, and incentives, whilst accounting for practical, primarily financial, constraints of food businesses. Alternative intervention approaches, targeting suppliers or customers, were suggested.

**Conclusions:**

Participants emphasised independent takeaways as particularly challenging, but worthwhile intervention targets. Participants perceived that interventions need to take account of the potentially challenging operating environment, particularly the primacy of the profit motive. Upstream interventions, engaging suppliers, as well as those that drive consumer demand, may be worth exploring. Rigorous, evidence-informed development and evaluation of such interventions is needed.

## Background

In the UK, the proportion of income spent on “eating out” increased gradually between the 1950s and mid-1980s [1]. Since the 1980s multinational fast-food restaurant and takeaway chains have expanded rapidly [2] and helped to create a culture of fast-food consumption [3]. In the UK these multinational chains share the market with a vibrant independent sector. The total UK spend on out-of-home eating was estimated as £73.05 billion in 2012, with £10.54 billion specifically on fast-food, takeaway and home-delivery products [4].

Meals from independent UK takeaways are high in total energy and salt when compared to UK Department of Health’s Dietary Reference Values [5]. A systematic review by Lachat et al. reported an association between ‘eating out-of-home’ and higher total energy intake [6]. Similarly, Summerbell et al., identified four prospective cohort studies in adults that found consumption of ‘fast-food’ was associated with higher levels of subsequent excess weight gain and obesity [7].

Proliferation of takeaway outlets, in the UK, is broadly concentrated in more deprived areas [8–10] and people living or commuting in close proximity to takeaways are more likely to be overweight or obese [11]. Therefore, takeaways are likely contributors to the obesogenic environment [12], specifically areas with a high density of unhealthy food outlets that have been termed ‘food swamps’ [13, 14].

In 2011 the UK government introduced the ‘Public Health Responsibility Deal’ which aimed to support and work with food businesses to develop and promote healthier food through voluntary pledges [15]. Whilst many chain restaurants committed to specific pledges, the Responsibility Deal did not extend to independently owned food outlets. Local authorities have been charged with the responsibility of working with independently owned food outlets to increase the provision of healthy food choices [16]. In 2013, the UK Department of Health indicated that further work was required at a local level to encourage small and medium sized enterprises to make changes that would support staff and help customers to make healthier choices [17]. Resources for owners and or managers of food outlets and local authorities have been provided to support this intention [17–19].

Multi-component voluntary schemes, such as healthy catering award schemes run by local authorities to increase the provision of healthy food choices [20], have been delivered across England [21]. The implementation of such schemes has principally been the responsibility of environmental health officers (EHO), with support from nutrition experts and public health improvement staff [21]. Our formative research that systematically identified and described interventions to promote healthier food in independent outlets in England has shown that, apart from Bagwell’s work [20], there is little evidence with regards to feasibility, acceptability or effectiveness [21]. Previous work has explored the reasons behind the difficulties and reluctance of food outlet vendors to offer healthier food [22], particularly in more deprived areas [20]. However, there is limited research on the experiences of those delivering interventions in their engagement with the food outlets and implementation of the intervention [20]. We therefore aimed to elicit the views of people with experience of delivering these types of interventions in independent food outlets to identify the operational challenges and their suggestions for best practice.

## Methods

We undertook individual, one-to-one, semi-structured qualitative interviews with employees of, or professionals contracted by, a local authority, who had experience of working with independent food outlets, including takeaways, to improve the healthiness of their food offering. We asked participants for their views on food available from takeaways, pubs, cafes, and restaurants, and their experience of delivering interventions in these settings.

### Participants and recruitment

We used purposive sampling [23] to recruit men and women, from a range of professional roles (public health, local authority enforcement officers and people with specific remits related to nutrition) from across different areas of England. Participants were professionals who had experience of working with owners and managers of independent takeaways, pubs, cafes, or restaurants to improve the healthiness of food on sale. Initial contacts were identified from prior work to map and synthesise evidence on interventions in out-of-home food outlets [21], with additional contacts recruited through snowball sampling [24]. We contacted people either by telephone or email to invite their participation in this study.

### Interviews

We used the findings of our recent systematic review on interventions in out-of-home food outlets [25] along with provisional discussions with intervention deliverers to develop an initial topic guide (see Appendix A), which was developed as the interviews progressed to allow for detailed comment on specific subjects. The topic guide covered: participants’ perceptions of takeaway food from independent out-of-home food outlets; participants’ experience of intervention deliverer; issues to consider for intervention design; and views on existing interventions. Interviews focussed on participants’ perceptions of the nutritional value of food from independent takeaways, pubs and restaurants, and their experience of intervention development and delivery. We planned to continue interviews until no new themes emerged [26]. Each participant was interviewed individually by one researcher (either LG or LP) either: face-to-face at the participant’s place of work; or by telephone and were digitally audio recorded.

### Analysis

Interviews were transcribed verbatim, read and checked against audio recordings for accuracy, and then anonymised. Framework analysis with constant comparison guided thematic data analysis [27] and was facilitated by the use of NVivo version 10 software [28, 29]. LG, LP and AAL read all interview transcripts. A coding framework was based on a priori themes from the topic guide and emergent themes that arose from the data. Coding of transcripts was conducted by LG, with independent coding of a subset of transcripts by LP. In regular meetings LG, LP and AAL discussed and agreed on the coding framework, identifying and further developing the themes and sub-themes. The coding framework was applied to all transcripts and the resulting themes and sub-themes were reviewed and agreed upon by all team members. Anonymised quotes are provided to illustrate themes.

## Results

We had a sample of 16 potential participants. Eleven interviews were conducted with no new themes emerging in later interviews. Interviews lasted 30 to 90 min. Eight participants were local authority enforcement officers working across environmental health (environmental health officers), trading standards or public protection (participants B-E, G-J), two had specific remits related to nutrition (A & F) and one was a health and wellbeing coordinator (K). All of the participants were either employees of, or contracted by, a local authority for the duration of their intervention work. Participants worked across ten different local authorities in England. Details regarding participants’ occupations and intervention experience can be found in Table 1. The topic guide developed as the interviews progressed to reflect the emergent themes. These included an increased focus on independent takeaways, the specific nutritional issues of concern, greater detail regarding intervention evaluation, and their consideration of different intervention methods.

**Table 1:** Participant details

Participant ID	English region	Gender	Occupation	Intervention experience
A	London	Male	Public health commissioning manager & registered nutritionist	Healthy catering award, targeted interventions
B	North-East England	Male	Environmental health and trading standards manager	Food sampling, healthy catering award, targeted interventions
C	North-East England	Male	Environmental health team leader	Takeaway food sampling
D	North-East England	Female	Senior environmental health officer	Healthy catering award
E	North-West England	Male	Environmental health officer	Chef, school meals area manager, cooking skills trainer, healthy catering awards, targeted interventions
F	South-West England	Female	Independent nutrition consultant	Worked with a range of independent catering businesses and schools
G	North-East England	Male	Principal environmental health officer	Food sampling, healthy catering award
H	North-East England	Male	Senior environmental health officer	Food sampling, healthy catering award
I	West Midlands	Male	Public protection officer	Healthy eating awards, targeted interventions
J	North-West England	Female	Trading standards officer	Healthy eating awards, targeted interventions
K	Yorkshire & The Humber	Female	Local authority project lead on nutrition training	Healthy eating awards, targeted interventions

### Food business operator

During initial interviews, intervention deliverers used a specific technical term, ‘food business operator’ (FBO). This person is defined as the natural or legal person responsible for ensuring that the requirements of food law are met within the food business under their control [30]. In most cases this person refers to either the owner or manager of the food outlet. We adopt this term throughout.

### Key findings

Our analysis identified a number themes and sub themes. These included: the perceived nature of unhealthy food offerings from independent takeaways; approaches to intervention; barriers and facilitators to interventions, including resourcing and legislation, authority and motivation of intervention deliverers, suppliers of ingredients and packaging, FBO characteristics and their local competition, and customers and community; and evaluation and impact of interventions. These are presented in detail below.

### The perceived nature of unhealthy food offerings from independent takeaways

Participants universally expressed condemnation of the nutritional quality, or lack of ‘healthiness’ of the food provided by independent takeaways where particular problems included excessive portion size, and high levels of saturated fat and salt.

*You’ve just got to look at the size of portions you get from a takeaway and they’re just ridiculous.* [D]

*One meal would give a woman three and a half days’ supply of her saturated fat allowance* [B]

Salt, in particular, was seen as a concern, specifically, high levels of salt in food from fish and chips shops was identified as a challenge. Sugar was seen as less of a concern, with the exception of sugar sweetened beverages available within all takeaways. However, certain cuisines were regarded as having higher levels of sugar, for example Chinese sweet and sour sauces. Fruit and vegetable content was perceived as low in most independent takeaway food, apart from in Chinese cuisine.

### Approaches to intervention

Within England, Northern Ireland and Wales a Food Hygiene Rating Scheme run by local authorities provides guidance to customers on the standard of food hygiene within all food businesses. Every business receives a rating from zero to five following an inspection by a food safety officer [31], with zero indicating ‘Urgent improvement necessary’ and five indicating ‘Very good’. Choosing independent takeaway outlets that had achieved a minimum rating of three, ‘Generally satisfactory’, was used as a benchmark for attempting intervention delivery with a business as it was perceived that independent takeaways that did not meet satisfactory hygiene requirements were unlikely to improve the nutritional quality of their food.

One participant suggested that, they perceived there was an underlying assumption on the part of the FBO, that working with the local authority environmental health team would be advantageous due to the EHO’s regulatory role.

*They generally know that if we’re supportive of their efforts then we will either (a) give them less of a hard time and (b) perhaps give them some degree of publicity and additional support.* [C]

Interventions identified by participants fell into three binary categories: covert or overt (to the consumer); single-target or multi-component; intensive or light-touch. Covert interventions, were those that were *not obvious* to the customer, which included, for example, cooking or recipe changes, such as skimming the fat off a dish during cooking or using less of a stronger tasting cheese. Covert (to the consumer) interventions were perceived to be more feasible by the participants because they did not rely on customer choice.

*We’ve been doing… sort of health by stealth, you know… talk about invisible changes that you’re making* [I]

Overt (to the consumer) interventions included providing information to customers to support healthier choices, such as menu labelling (e.g. identifying an item as lower in fat).

Single-target interventions, with one nutritional focus, were rare. However, there was one widely cited example that involved replacing the 17-hole cap of a counter-top salt shaker with a 5-hole cap to reduce the amount of salt dispensed [32, 33]. Multi-component interventions were more common, especially healthy catering award schemes. Such schemes required a food outlet to meet a range of criteria in order to receive an award. Some considered that achieving these multiple targets to obtain an award was a barrier to success.

*It [the award] was lots of criteria… which is the limiting factor in [name of local authority] because an award that maybe awarded you for having no salt, and low sugar might be more effective than saying “we want you to meet all these criteria.”* [D]

Resource intensive interventions, such as FBO engagement workshops, were thought to be more successful than light-touch approaches, such as advice leaflets.

*We could have maybe gone down the route of leaflets and one-off stuff which I think would have been a waste.* [G]

Participants reported that they believed healthy catering award schemes did not offer valued incentives to independent takeaways and therefore were not sufficiently motivational to FBOs in order to achieve change. By contrast, the 5-hole salt shaker was promoted to FBOs by detailing the minimal impact on business practices and potential financial savings that could be made from delivering less salt. Interventions that sought to support businesses through advertising and contributions to specific business costs of offering healthier foods, such as updating menus, were perceived as feasible options to support FBOs in implementing a change towards provision of healthier food.

*If somebody had said, “Well, I’ve just had 2000 menus printed and I wouldn’t be due to do them again for another year or two and you want me to highlight the healthy options.” Well, I would have said to them, “How much does it cost and we’ll pay, we’ll give 50% towards it.”* [K]

Participants expressed a strong belief that any intervention must take account of the competitive business environment and constraints in which the FBO worked.

*…a businessman would say “well what’s in it for me?”* [E]

This included delivering the intervention at a time that was acceptable to the FBO.

*We offered them three different [branded workshop] dates and we set each one at a different time* [K]

Participants detailed effective strategies for intervention delivery such as simple, clear, step-by-step instructions, tailored to the specific cuisine type that could be easy to implement. Interventions that included practical engagement, such as interactive tasting sessions, were seen as particularly engaging.

*…what really went down well is we actually did a taste test as part of our [branded workshop].* [K]

Participants stated that, regardless of the intervention method, it was important to develop a strong working relationship with the FBO and their business.

*…if you build the relationship up, you know, I’ll literally go in the kitchen and show them how to do something that they’ve said they can’t do, I’d say “you can, I’ll show you.” And you’ll go and do that and they’ll think “oh right, I can, that’s cheaper to do it that way.”* [E]

Participants also emphasised how important it was to acknowledge FBOs for the efforts they had made to change.

*We’re about saying “look, let’s build on the positive things that you’re doing and perhaps introduce some healthier options, and in the meantime we might just increase the reputation of your business and your profits”* [E]

### Barriers and facilitators to interventions

**Fig. 1:**
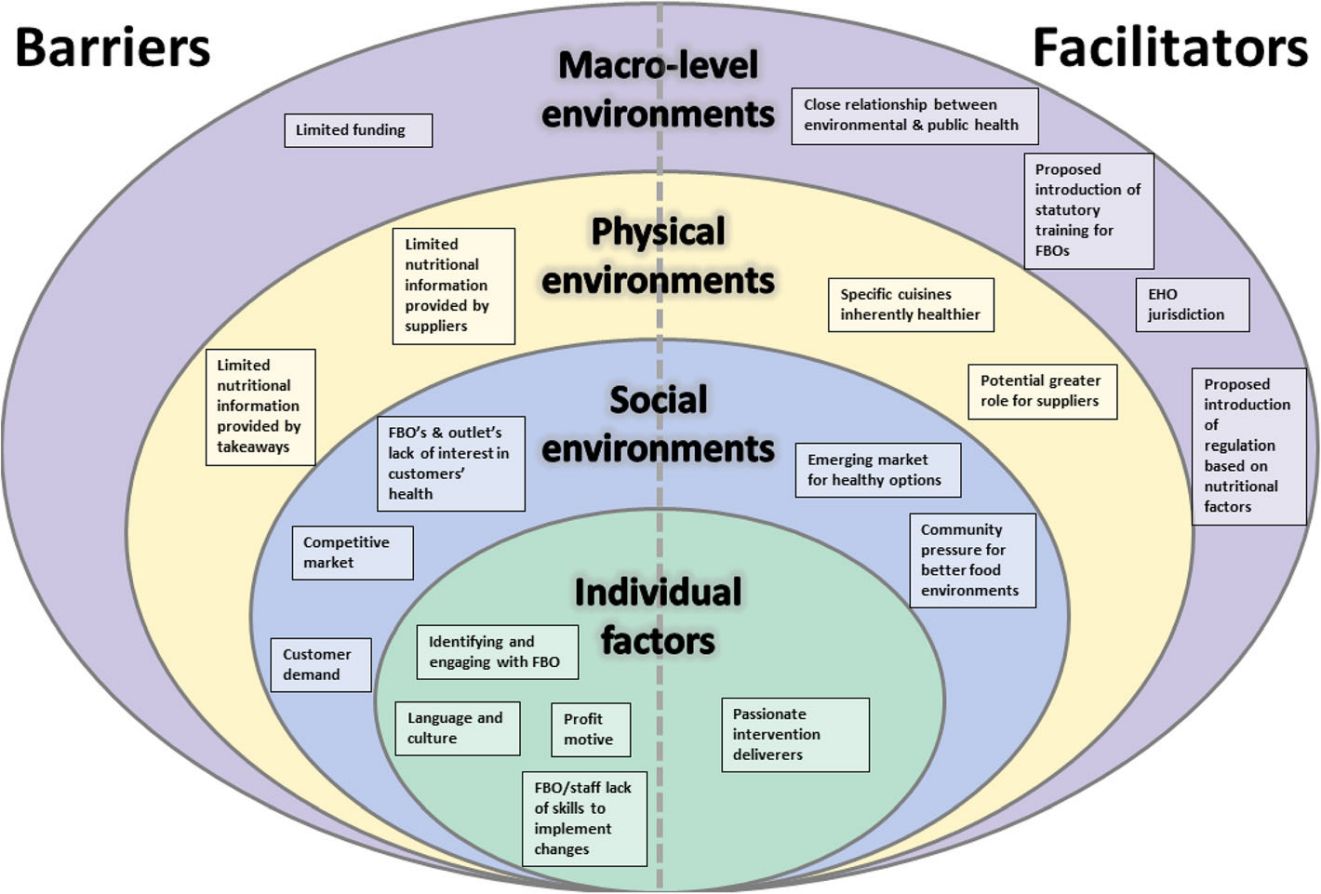
Barriers and facilitators to delivering interventions to promote healthier food mapped to Story et al.’s [34] ecological framework

Participants identified barriers and facilitators to intervention delivery across five operational levels. Additionally, each has been mapped to an adapted version of Story et al.’s ecological framework which describes the multiple influences on food availability [34]. This adapted framework depicts factors that are barriers and facilitators to delivering interventions to promote healthier food. They include macro-level barriers (e.g. limited funding) and facilitators (e.g. EHO jurisdiction) through to individual barriers (e.g. language and culture) as well as individual facilitators (e.g. passionate intervention deliverers) (Fig. 1).

#### Resourcing and legislation

Most participants were employed directly by a local authority. They spoke about changes to their work structure that brought about a closer working relationship with public health officials who had an interest in targeting independent takeaways.

*…public health very much want me to target takeaways and that’s what their big thing is, you know the takeaway outlets.* [E]

Limited funding, compounded by recent cuts in local authority budgets, was the main barrier to intervention delivery.

*We try and do what we can but when your resources are dwindling constantly and you’ve got other pressures on your time, it’s not easy.* [J]

With regard to legislation that could facilitate improvements, participants suggested a statutory training requirement for FBOs and their staff, including nutritional education.

*If you even run a pub you have to have a licence to run a pub. The situation at the moment is that anybody can open a food business, they don’t have to have any qualification when they open that business, they don’t have to have a licence, they don’t have to have a cooking qualification for instance.* [E]

Others proposed a tax on fat or regulation of portion size. Participants felt there was legislative scope to restrict the opening of new independent takeaways, but emphasised that such changes would have no impact on existing establishments.

*We’ve just had our supplementary planning document ratified and so we are going to be able to [restrict] the proliferation of these types of outlets, but the issue is that only stops the new ones, it doesn’t do anything about the existing businesses.* [J]

#### Authority and motivation of intervention deliverers

For some participants nutrition related intervention work was additional to their statutory duties, while others were contracted specifically to conduct this work. Many participants expressed their passion for public health improvement particularly within their local area and community as a personal motivation to facilitate intervention delivery.

*I would love to get more involved, it’s my passion. Food’s my passion. And then I see unhealthy kids all the time and you just think “oh we should be doing, this shouldn’t be happening”* [E]

Those participants who were employed directly by a local authority identified the authority of an EHO as important in gaining initial access to certain independent takeaways.

*…you’re going in there with the badge of an EHO in the hope that, you know, the staff or the owner will be engaging because, because of that EHO relationship.* [K]

#### Suppliers of ingredients and packaging

All of the approaches to interventions discussed in interviews were targeted at changes within individual independent takeaways. However, some participants identified the role played by independent takeaway suppliers (wholesalers) as important and felt that there was potential to engage with them to deliver changes, such as provision of ‘*healthier*’ alternatives, which would provide individual independent takeaways with the option to purchase such products.

*…they [the suppliers] could more clearly show businesses that there are other options, you know the sort of [healthy] varieties which are the same price… that would be useful.* [A]

It was suggested that by limiting the types, cost and size of products and packaging, suppliers could directly influence food and portion sizes offered to customers.

One participant took issue with the way suppliers labelled their products, implying customers were deliberately misled.

*The national suppliers are very good at obfuscation of the nutritional value of their food.* [B]

The few participants who had tried to engage with suppliers about provision of ‘*healthier alternatives*’ had received mixed responses, but there was broad agreement that consideration should be given to the suppliers’ role in independent takeaway food provisions.

#### FBO characteristics and their local competition

All participants identified the need to engage with FBOs, as they were of the view that FBOs have the greatest influence over menus at their establishments.

*…even if the people actually doing the cooking are really into [healthier food], if the manager hasn't bought into it, then it's quite difficult for them to actually do it* [F]

However, establishing contact with FBOs was often reported as difficult. Participants reported that FBOs were frequently evasive and might try to avoid having their name on official documentation.

*…sometimes it is actually very difficult to identify who the actual business owner is… because nobody seems to take ownership.* [J]

Language or culture was also stated as a barrier to engagement. Participants reported that English was often not the first language of the FBO or their staff.

*You could speak to the owner and they would say that “my staff don’t speak English very well, it’s too much work for me to explain it all to them” and you know there was a lot of resistance.* [J]

Participants also recognised that profit was the top priority for FBOs.

*…a business's bottom line is to make profit* [K]

Independent takeaways were perceived by participants to operate in a highly competitive market with high business turnover and low profit margins. This focus on profit and consequently customer numbers meant that issues regarding the ‘*healthiness*’ of food were not seen as a key concern. As a result of intense competition, participants reported that one of the main marketing strategies used by independent takeaway outlets was to offer larger portions at a lower prices, focussing on ‘*value-for-money*’.

*They were very fixated with people wanting value-for-money, and that's why they pile the food on.* [F]

This was coupled with a perception by participants that FBOs were disinterested in the health of their customers.

*They’re not really thinking about what the customers actually could need in terms of health* [G]

This did not mean that there were no healthy options within the independent takeaway sector. Some participants were keen to point out what they thought of as healthier examples.

*The Cantonese style food, they tend to be much more aware [of healthiness of their product] mainly because of the upbringing they’ve have and the food they’ve had when growing up they tend to be more aware* [J]

*There’s big improvements with the charcoal based cuisine places at the moment because that’s to do with the big surge in popularity of say the [national grilled chicken restaurant chain] type products, so piri-piri chicken, grilled chicken, barbecue chicken* [E]

Where FBOs were willing to participate in an intervention, participants sometimes reported a lack of knowledge or skills to promote their healthier options. However, some participants reported positive experiences such as working with engaged FBOs to identify methods to advertise positive changes to their customers.

*Some of them were so up for it. We actually had to produce a poster saying we are now giving less salt on your food* [B]

But such enthusiasm was not universal.

*We do provide them with a poster about the reduced salt [and] the salt shaker, which some of them put up, some of them didn’t.* [I]

#### Customers and community

Participants universally reported that, in their view, customers chose takeaway food because it provided a cheap, convenient and filling meal. One expressed a view that ‘*price discounting*’ encouraged customers to consume takeaway food multiple times throughout the week. However, some participants perceived there was an emerging market for healthier food options.

*So you’ve got that age group of males, who are more frequently asking for lean products, grilled products, chicken predominantly… you know they’ve all got apps on their phone now to tell them how many calories are in things.* [E]

It was widely acknowledged that independent takeaway outlets did not make it easy for customers to identify the healthier options. There was little calorie or nutrient information provided in independent takeaway outlets.

There was a strong feeling of customer demand driving the takeaway market.

*The fact that they’ve [the FBOs] got customers coming through the door is their main problem. If they saw a reduction in customers, they would need to think about how to diversify.* [A]

Participants also suggested that interventions needed to take account of customers’ taste preferences. For example, salt reduction was considered problematic because of its direct link to taste.

*The difficult thing on salt is people will associate it with flavour of course and if they think you are offering food with less flavour, that’s the barrier.* [H]

Community pressure was considered a potential driver of change.

*I think we need to approach this in a more community-based way to involve communities and community-leaders, and you know schools, churches, anything like that to try to create a community ownership if you will, rather than the government saying “you cannot do this.”* [E]

Therefore, identifying and targeting the right people and institutions, with the greatest influence within communities, was seen as critical to create a feeling of ‘*community ownership’*.

*I’ve built up a lot of relationships, a lot of them managers with independent businesses, private traders, all the Sure Start centres… All those people ring up for advice. They’ll ask me to go and speak to parent groups about healthy food. I’m very involved with schools… we need… to involve communities and community-leaders, and you know schools, churches, anything like that to try to get some community, to create a community ownership.* [E]

### Evaluation and impact of interventions

Participants saw their work as service delivery – evaluation was not considered essential within the local authority setting.

*We’re more, go in and do the work, what we’ve not been great at or not had the capacity to do [is evaluation]* [K]

Factors contributing to the lack of evaluation included limited resources, the constraints and demands of the local authority working environment and a lack of knowledge and skills regarding how to conduct evaluation. Some participants suggested evaluation should be carried out independently.

*I always think evaluation, where possible, should be independent anyway so that you, you’re less likely to have skewed reporting* [K]

All participants acknowledged that independent takeaways, in particular, represented a challenging setting and some were sceptical regarding intervention impact.

*You can get them to do small things but whether they [the changes made] have a massive impact is debateable.* [J]

However, participants appeared united that work to improve the healthiness of independent takeaway food was important and further efforts should be made.

*People have been doing this kind of health promotion work for a long time now, and [local authority] health stats are still appalling. So can you make a massive difference to a landscape? I’m not sure. But can you get some wins? …I think you’ve got to try haven’t you? You can’t just let it go on as it is* [H]

## Discussion

### Summary of principal findings

To our knowledge, this is the first study to explore the views of people with experience of delivering interventions to promote healthier independent takeaway food via a range of interventions across England. The results highlight the challenging nature of delivering such interventions. Participants identified independent takeaways as offering predominantly unhealthy food, with large portion size and high levels of saturated fat and salt. They described a range of approaches to intervention delivery that they perceived as influencing intervention effectiveness and acceptability to FBOs. We categorised these as: covert or overt (to the consumer), single-target or multi-component, intensive or light-touch. Healthy catering award schemes (overt and multi-component) were perceived by participants as largely ineffective. Factors facilitating effective intervention delivery were perceived to be: intensive and interactive programmes (with the FBO), provision of step-by-step instructions, awareness of financial considerations and minimal disruption to usual business practices. Building strong, respectful relationships between FBOs and intervention deliverers was seen as key for success. We identified barriers to successful intervention delivery at a number of operation levels: availability of resources, both financial and workforce, and willingness of independent takeaway FBOs to engage with interventions.

### Strengths and limitations of the methods

This qualitative study has provided in-depth contextual information regarding the practicalities of delivering nutritional interventions targeted at independent takeaway outlets.

Participants were identified from prior work, a systematic mapping and evidence synthesis of interventions promoting healthier ready-to-eat meals (to eat in, to take away, or to be delivered) sold by specific food outlets in England [21]. This resulted in inclusion of participants from ten different local authorities, representing a range of views of intervention delivery experience. At 11 interviews no new themes emerged, but it is possible that this is not a representative sample and therefore does not represent all views.

Interviews focused on interventions in small, independent takeaways, and therefore may not be generalizable beyond England and further it is unlikely that the findings are generalizable to larger enterprises, chains or franchises, where management structure and responsibility for the food offerings will differ substantially.

### Comparison with other studies

The findings from these interviews suggest that intervention deliverers believe that multi-component catering award schemes have been broadly ineffective at recruiting independent takeaway businesses. This reflects the findings of recent work with independent fast food vendors in Scotland [22].

Our findings, suggests that FBOs may feel restricted in their ability to offer healthier food due to the market pressures under which they operate, reflecting previous work [20, 22]. Bagwell [20] explored the views of intervention deliverers in relation to the London based Healthy Catering Commitment, an award style intervention, that aimed to get small independent outlets to adopt healthier cooking practices and reported similar factors influencing the level of business commitment to such initiatives as we found. These included operational barriers, issues around the limitations of suppliers, aspects of the local trading environment such as differing cultural practices and the actual or perceived impact of the changes on profits. Similar to the perception of our participants, businesses in London also preferred less intrusive (to the customer) interventions. Our study adds further context in relation to resourcing interventions and constraints at the supply level. Our work highlights the multiple layers of influence on the food that is served within independent takeaways, and suggests the need to adopt a multi-level, socio-ecological approach to designing interventions in this complex setting [34]. In this context it is plausible that interventions either upstream (i.e. with suppliers) or downstream (i.e. with customers) of independent takeaways may be more effective than those directly targeting independent takeaway FBOs themselves.

### Implications

Our work suggests that interventions specifically tailored to particular settings may be more effective than more generic approaches. Our findings highlight the practical challenges of developing and implementing interventions to promote healthier food offerings in independent takeaways. We have identified intervention components that intervention deliverers associate with effective delivery, feasibility and acceptability to FBOs. It should be noted that this does not include evaluation of effectiveness. Future intervention development could build on our findings regarding potential feasibility. For example, there should be a focus on covert interventions that deliver nutritional changes that are not immediately obvious to the customer, as these were perceived by intervention deliverers to be most feasible and acceptable to FBOs. Future interventions should also include a measure of effectiveness.

By contrast overt healthy catering award schemes were perceived as difficult to implement and unlikely to be effective. Once funding has been secured for an intervention the main focus should be on FBO engagement and building a mutual relationship of trust; this can be time consuming, but appears to be important for successful implementation. Our findings also suggest that it is critical to consider the financial implications of interventions for businesses due to tight profit margins and the competitive business environment in which they operate. Further, our study also suggests that future intervention work should look at the potential of *both* upstream and multi-level approaches such as working with suppliers or regulators and creating customer demand or local competition for healthier food choices in independent takeaways. If addressed collectively such changes may be more effective in driving the provision of healthier food within independent takeaway outlets.

### Unanswered questions and future research

Future work should look for opportunities to develop, deliver and evaluate covert (to the consumer) interventions. The 5-hole salt shaker is one such intervention, which we recently evaluated [32, 33]. We concluded that their use is associated with lower relative sodium content of fish and chip meals [32]. A limitation of this intervention technology is that it is linked to specific takeaway cuisines and to salt added at point of sale.

The 5-hole salt shaker relies on technology, but other covert (to the consumer) intervention opportunities, such as variation in recipes or cooking methods to reduce the fat or salt content of meals, require a greater level of FBO engagement. Offering training to FBOs to facilitate healthier meal preparation may appear to be an attractive option, but can be resource intensive and should be evaluated for likely benefit in relation to cost. Increased consideration should be given as to how to engage with FBOs and their staff. Interactive forums, such as workshops, where FBOs and other stakeholders are able to meet collectively to discuss issues regarding health in a non-judgmental setting, could be explored.

The participants in this study identified opportunities to consider engagement with stakeholders, other than the FBOs, such as independent takeaway customers and suppliers of ingredients and packaging to independent takeaways. One potential intervention target, which would be applicable across all independent takeaway cuisine types, is portion size. Packaging exists, for some foods that restricts portion size, such as cardboard or polystyrene boxes [35]. Thus suppliers could have a role in developing and delivering packaging that would support independent takeaway business in reducing portion size, or offering a wider choice of portion sizes.

This research lacks the voices of other key stakeholders, such as policy makers, intervention commissioners and customers. Future work could also explore how independent takeaway customers can be mobilised to increase demand for healthier options, as FBOs are more likely to be responsive to customers than any other stakeholder group.

With all future work, there is a need for rigorous theory and evidence-informed development and evaluation of interventions in order to guide future efforts in this area.

## Conclusions

Participants delivering interventions in independent takeaways found such work challenging. However, they stressed that independent takeaways (despite the challenges) were important targets for change due to their nutritionally poor food offerings. Interventions need to take account of the potentially challenging operating environment, particularly the primacy of the profit motive. Participants suggested that future work should explore the possibility of utilising suppliers or customers as the agents of change. Our findings on the different operational levels at which interventions can be applied and their associated barriers and facilitators, together with the advantages and disadvantages of intervention delivery methods (covert or overt (to the consumer), single-target or multi-component, intensive or light touch) can be used to inform future intervention development.

## Abbreviations

EHO: Environmental health officer
FBO: Food business operator
